# Four Novel Loci (19q13, 6q24, 12q24, and 5q14) Influence the Microcirculation *In Vivo*

**DOI:** 10.1371/journal.pgen.1001184

**Published:** 2010-10-28

**Authors:** M. Kamran Ikram, Sim Xueling, Richard A. Jensen, Mary Frances Cotch, Alex W. Hewitt, M. Arfan Ikram, Jie Jin Wang, Ronald Klein, Barbara E. K. Klein, Monique M. B. Breteler, Ning Cheung, Gerald Liew, Paul Mitchell, Andre G. Uitterlinden, Fernando Rivadeneira, Albert Hofman, Paulus T. V. M. de Jong, Cornelia M. van Duijn, Linda Kao, Ching-Yu Cheng, Albert Vernon Smith, Nicole L. Glazer, Thomas Lumley, Barbara McKnight, Bruce M. Psaty, Fridbert Jonasson, Gudny Eiriksdottir, Thor Aspelund, Global BPgen Consortium, Tamara B. Harris, Lenore J. Launer, Kent D. Taylor, Xiaohui Li, Sudha K. Iyengar, Quansheng Xi, Theru A. Sivakumaran, David A. Mackey, Stuart MacGregor, Nicholas G. Martin, Terri L. Young, Josh C. Bis, Kerri L. Wiggins, Susan R. Heckbert, Christopher J. Hammond, Toby Andrew, Samantha Fahy, John Attia, Elizabeth G. Holliday, Rodney J. Scott, F. M. Amirul Islam, Jerome I. Rotter, Annie K. McAuley, Eric Boerwinkle, E. Shyong Tai, Vilmundur Gudnason, David S. Siscovick, Johannes R. Vingerling, Tien Y. Wong

**Affiliations:** 1 Department of Epidemiology, Erasmus Medical Center, Rotterdam, The Netherlands; 2 Department of Ophthalmology, Erasmus Medical Center, Rotterdam, The Netherlands; 3 Department of Neurology, Erasmus Medical Center, Rotterdam, The Netherlands; 4 Centre for Molecular Epidemiology, Yong Loo Lin School of Medicine, National University of Singapore, Singapore; 5 Cardiovascular Health Research Unit, University of Washington, Seattle, Washington, United States of America; 6 Department of Epidemiology, University of Washington, Seattle, Washington, United States of America; 7 Division of Epidemiology and Clinical Applications, National Eye Institute, Intramural Research Program, National Institutes of Health, Bethesda, Maryland, United States of America; 8 Centre for Eye Research Australia, University of Melbourne, Royal Victorian Eye and Ear Hospital, Melbourne, Australia; 9 Centre for Vision Research, Department of Ophthalmology and the Westmead Millennium Institute, University of Sydney, Sydney, Australia; 10 Department of Ophthalmology and Visual Science, University of Wisconsin, Madison, Wisconsin, United States of America; 11 Department of Internal Medicine, Erasmus Medical Center, Rotterdam, The Netherlands; 12 Department of Clinical Chemistry, Erasmus Medical Center, Rotterdam, The Netherlands; 13 Netherlands Institute of Neuroscience, Amsterdam, The Netherlands; 14 Department of Ophthalmology, Academic Medical Center, Amsterdam, The Netherlands; 15 Department of Epidemiology, Johns Hopkins University Bloomberg School of Public Health, Baltimore, Maryland, United States of America; 16 Department of Ophthalmology, Taipei Veterans General Hospital, Taipei, Taiwan; 17 Department of Ophthalmology, National Yang-Ming University School of Medicine, Taipei, Taiwan; 18 Icelandic Heart Association, Kopavogur, Iceland; 19 Faculty of Medicine, University of Iceland, Reykjavik, Iceland; 20 Department of Medicine, University of Washington, Seattle, Washington, United States of America; 21 Department of Biostatistics, University of Washington, Seattle, Washington, United States of America; 22 Department of Health Services, University of Washington, Seattle, Washington, United States of America; 23 Center for Health Studies, Group Health, Seattle, Washington, United States of America; 24 Department of Ophthalmology, University of Iceland, Reykjavik, Iceland; 25 Department of Ophthalmology, Landspitalinn University Hospital, Reykjavik, Iceland; 26 Department of Statistics, University of Iceland, Reykjavik, Iceland; 27 Laboratory of Epidemiology, Demography, and Biometry, National Institute on Aging, Intramural Research Program, National Institutes of Health, Bethesda, Maryland, United States of America; 28 Medical Genetics Institute, Cedars-Sinai Medical Center, Los Angeles, California, United States of America; 29 Department of Epidemiology and Biostatistics, Case Western Reserve University, Cleveland, Ohio, United States of America; 30 Lions Eye Institute, University of Western Australia, Centre for Ophthalmology and Visual Science, Perth, Australia; 31 Genetics and Population Health, Queensland Institute of Medical Research, Brisbane, Australia; 32 Center for Human Genetics, Duke University Medical Center, Durham, North Carolina, United States of America; 33 Cardiovascular Health Research Unit, Department of Medicine, University of Washington, Seattle, Washington, United States of America; 34 Cardiovascular Health Research Unit, Department of Epidemiology, University of Washington, Seattle, Washington, United States of America; 35 Center for Health Studies, Group Health, Seattle, Washington, United States of America; 36 Department of Twin Research and Genetic Epidemiology, King's College London School of Medicine, St Thomas' Hospital, London, United Kingdom; 37 School of Biomedical Sciences, University of Newcastle, Callaghan, Australia; 38 Hunter Medical Research Institute, Newcastle, Australia; 39 Human Genetics Center and Institute of Molecular Medicine, University of Texas Health Science Center at Houston, Houston, Texas, United States of America; 40 Department of Epidemiology and Public Health, Yong Loo Lin School of Medicine, National University of Singapore, Singapore; 41 Department of Medicine, Yong Loo Lin School of Medicine, National University of Singapore, Singapore; 42 Singapore National Eye Centre and Singapore Eye Research Institute, Singapore; 43 Department of Ophthalmology, Yong Loo Lin School of Medicine, National University of Singapore, Singapore; University of Oxford, United Kingdom

## Abstract

There is increasing evidence that the microcirculation plays an important role in the pathogenesis of cardiovascular diseases. Changes in retinal vascular caliber reflect early microvascular disease and predict incident cardiovascular events. We performed a genome-wide association study to identify genetic variants associated with retinal vascular caliber. We analyzed data from four population-based discovery cohorts with 15,358 unrelated Caucasian individuals, who are members of the Cohort for Heart and Aging Research in Genomic Epidemiology (CHARGE) consortium, and replicated findings in four independent Caucasian cohorts (n = 6,652). All participants had retinal photography and retinal arteriolar and venular caliber measured from computer software. In the discovery cohorts, 179 single nucleotide polymorphisms (SNP) spread across five loci were significantly associated (p<5.0×10^−8^) with retinal venular caliber, but none showed association with arteriolar caliber. Collectively, these five loci explain 1.0%–3.2% of the variation in retinal venular caliber. Four out of these five loci were confirmed in independent replication samples. In the combined analyses, the top SNPs at each locus were: rs2287921 (19q13; p = 1.61×10^−25^, within the *RASIP1* locus), rs225717 (6q24; p = 1.25×10^−16^, adjacent to the *VTA1* and *NMBR* loci), rs10774625 (12q24; p = 2.15×10^−13^, in the region of *ATXN2,SH2B3* and *PTPN11* loci), and rs17421627 (5q14; p = 7.32×10^−16^, adjacent to the *MEF2C* locus). In two independent samples, locus 12q24 was also associated with coronary heart disease and hypertension. Our population-based genome-wide association study demonstrates four novel loci associated with retinal venular caliber, an endophenotype of the microcirculation associated with clinical cardiovascular disease. These data provide further insights into the contribution and biological mechanisms of microcirculatory changes that underlie cardiovascular disease.

## Author Summary

The microcirculation plays an important role in the development of cardiovascular diseases. Retinal vascular caliber changes reflect early microvascular disease and predict incident cardiovascular events. In order to identify genetic variants associated with retinal vascular caliber, we performed a genome-wide association study and analyzed data from four population-based discovery cohorts with 15,358 unrelated Caucasian individuals, who are members of the Cohort for Heart and Aging Research in Genomic Epidemiology (CHARGE) consortium, and replicated findings in four independent Caucasian cohorts (n = 6,652). We found evidence for association of four loci with retinal venular caliber: on chromosomes 19q13 within the *RASIP1* locus, 6q24 adjacent to the *VTA1* and *NMBR* loci, 12q24 in the region of *ATXN2,SH2B3* and *PTPN11* loci, and 5q14 adjacent to the *MEF2C* locus. In two independent samples, locus 12q24 was also associated with coronary heart disease and hypertension. In the present study, we demonstrate that four novel loci were associated with retinal venular caliber, an endophenotype of the microcirculation associated with clinical cardiovascular disease. Our findings will help focus research on novel genes and pathways involving the microcirculation and its role in the development of cardiovascular disease.

## Introduction

Although both macrovascular and microvascular pathology are associated with cardiovascular disease, including coronary artery disease and stroke [1], [2], most studies on the genetic determinants of cardiovascular disease have primarily focused on macrovascular disease traits, and genetic analyses of microvascular disease phenotypes are rare [2], [3]. This paucity of data is due to difficulties in non-invasively assessing the microcirculation. However, retinal arterioles and venules, which range between 50 to 300 µm in diameter, can be directly imaged, and provide an ideal opportunity to study the microcirculation *in vivo* [4].

Quantitative measurement of retinal blood vessel caliber from photographs allows a non-invasive direct assessment of the human microcirculation [4]. Studies using this technique have shown that changes in retinal vascular caliber (e.g., narrower arteriolar and wider venular caliber) are associated with a range of cardiovascular diseases and their risk factors [5], [6], including hypertension [7], diabetes mellitus [8], [9], stroke [10], coronary heart disease [11], and cerebral small vessel disease [12], [13]. Retinal vascular caliber is also an early marker of major eye diseases such as diabetic retinopathy and age-related macular degeneration [14]–[16].

Recent studies suggest that genetic factors may play a role in influencing retinal vascular caliber [17]–[20], so understanding specific genetic factors underlying retinal vascular caliber could therefore demonstrate novel insights into the mechanisms that contribute to the microvascular pathways of cardiovascular and eye diseases. To identify the underlying genetic determinants of retinal arteriolar and venular caliber, we meta-analyzed results of genome-wide association studies (GWAS) of 15,358 white participants from four large, prospective population-based cohorts included in the Cohorts for Heart and Aging Research in Genomic Epidemiology (CHARGE) consortium [21]: the Age Gene/Environment Susceptibility – Reykjavik Study (AGES) [22], the Atherosclerosis Risk in Communities Study (ARIC) [23], the Cardiovascular Health Study (CHS) [24] and the Rotterdam Study [25]. We replicated our findings in four independent cohorts of Caucasian ethnicity [the Australian Twins Study [26], the UK Twins Study [27], the Beaver Dam Eye Study (BDES) [11], and the Blue Mountains Eye Study (BMES)] [11]. Finally, in order to examine the association between the replicated hits and cardiovascular diseases, we used data on coronary artery disease from the Wellcome Trust Case Control Consortium (WTCCC) [3], on stroke and myocardial infarction from the Heart and Vascular Health (HVH) Study [28], [29], on hypertension from the Global Blood Pressure Genetics (Global BPgen) Consortium [30], and on diabetes mellitus from the Diabetes Genetics Replication and Meta-analysis + (DIAGRAM+) Consortium [31].

## Results

### Study samples

The total study sample for the discovery analyses was 15,358 and for the replication analyses 6,652. Characteristics of both the discovery and replication samples are presented in Table 1.

**Table 1:** Baseline characteristics of both the discovery and replication cohorts.

	Discovery cohorts				Replication cohorts			
	*AGES*	*ARIC*	*CHS*	*RS*	*Australian Twins*	*UK Twins*	*BDES*	*BMES*
Original cohort	5,764	15,792	5,888	7,983	2,235	8,810	2,579	3,508
Non-Hispanic whites in original cohort		11,478	4,925	7,983	2,190	8,810	2,579	3,487
Total number included in analyses	2,949	6,317	1,272	4,820	1,709	1,132	2,522	1,310
Mean age (years) (SD) [range]	76.2 (5.4)[66–94]	60.3 (5.6)[50–72]	78.4 (4.1)[72–95]	68.0 (8.2)[55–99]	22.5 (12.4)[5–90]	58.1 (10.1)[16–81]	60.6 (10.8)[43–86]	66.0 (8.6)[49–93]
Proportion female (%)	57.5	52.9	62.9	59.0	57.0	97.7	55.8	58.4
Mean CRAE (µm) (SD) [range]	139.7(13.4)[74.0–221.4]	136.1 (14.3)[72.6–203.8]	140.4 (15.7)[77.6–197.4]	150.0 (14.4)[98.5–235.4]	164.2 (13.6)[83.6–205.2]	163.8 (18.1)[91.0–219.6]	149.5 (13.7)[100.3–196.6]	160.0 (20.2)[93.4–213.4]
Mean CRVE (µm) (SD) [range]	202.0(19.5)[123.8–273.0]	199.4 (19.2)[129.3–304.1]	196.5 (19.2)[142.5–271.7]	226.0 (20.1)[162.5–324.3]	248.0 (19.0)[130.5–325.7]	253.0 (28.6)[147.0–338.0]	230.3 (21.7)[165.9–335.1]	238.4 (24.0)[167.6–331.1]
Systolic blood pressure (mm Hg) (SD) [range]	142.5(20.2)[92–253]	122.9 (18.1)[75–226]	134.4 (20.4)[82–241]	138.5 (22.1)[74–250]	N/A	130.5 (19.7)[85–210]	130.5 (20.0)[71–248]	146.0 (20.5)[95–240]
Diastolic blood pressure (mm Hg) (SD) [range]	74.1 (20.2)[92–253]	70.8 (10.0)[32–114]	67.9 (10.8)[15–110]	73.7 (11.4)[24–139]	N/A	79.5 (11.9)[50–124]	77.4 (10.8)[44–123]	83.6 (9.8)[50–125]
Hypertension (%)	80.6	35.3	48.7	42.3	3.2	41.7	35.1	46.1
Diabetes mellitus (%)	11.4	12.4	12.2	10.0	1.0	1.5	10.3	7.9
Current smokers (%)	12.5	17.3	6.2	23.6	11.0	13.8	21.6	12.4
Body mass index (kg/m^2^) (SD) [range]	27.1 (4.4)[14.8–48.5]	28.0 (5.2)[14.2–59.1]	26.8 (4.3)[15.6–46.7]	26.3 (3.7)[14.2–50.7]	N/A	25.6 (4.3)[15.0–48.2]	28.3 (5.2)[15–55]	26.3 (4.4)15.2–49.2

AGES: Age Gene/Environment Susceptibility – Reykjavik Study; ARIC: Atherosclerosis Risk in Communities Study; CHS: Cardiovascular Health Study; RS: Rotterdam Study; BDES: Beaver Dam Eye Study; BMES: Blue Mountains Eye Study; CRAE: central retinal arteriolar equivalent; CRVE: central retinal venular equivalent; SD: standard deviation; N/A Not available.

### Meta-analysis of CHARGE cohort results

**Figure 1:**
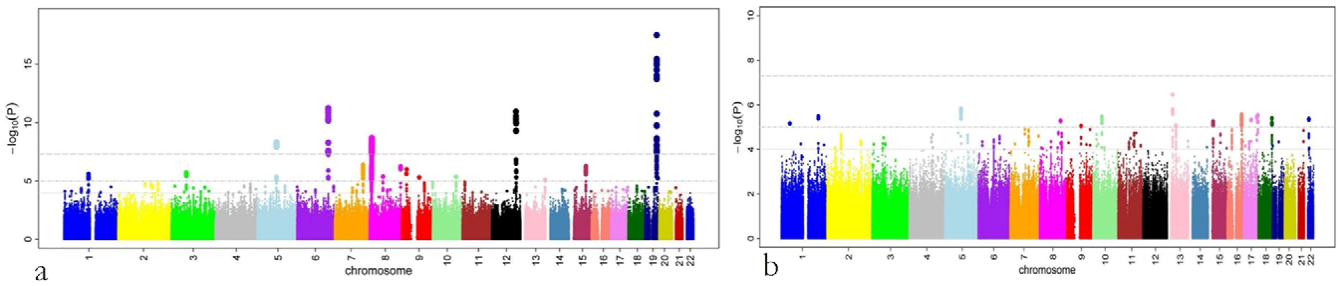
P-values (minus log-transformed) are shown in a signal intensity (Manhattan) plot relative to their genomic position. For (a) retinal venular caliber and (b) retinal arteriolar caliber.

A total of 179 single nucleotide polymorphisms (SNPs) at five loci surpassed our preset threshold (p<5.0×10^−8^) for genome-wide significance for retinal venular caliber. Collectively, these five independent loci explain 1.0–3.2% of the variation in retinal venular caliber within our discovery cohorts. The QQ-plots (Figure S1A) show departure from the line of identity at approximately p<1.0×10^−3^. Figure 1A displays the minus log-transformed p-values for the individual SNPs against their genomic position. Table 2 summarizes both the meta-analyzed results and results from each discovery cohort individually for the most significant SNPs at each locus that were associated with retinal venular caliber.

**Table 2:** Results for the five loci associated (p<5.0×10^−8^) with retinal venular caliber in the discovery cohorts both combined and individually.

	*Discovery cohorts combined*				*Discovery cohorts individually*														
	*CHARGE*				*AGES*			*ARIC*			*CHS*			*RS*					
SNP (chromosome position) locus	Minor allele (MAF)	Beta	SE	P-value	Beta	SE	P-value	Beta	SE	P-value	Beta	SE	P-value	Beta	SE	P-value	Genes of Interest	Other genes within 60kb	Additional SNPs at p-value<5×10^−8^
rs2287921 (53920084)19q13	T (0.47)	−2.0	0.23	3.30×10^−18^	−1.0	0.56	7.40×10^−2^	−2.5	0.36	3.80×10^−12^	−2.9	0.77	1.66×10^−4^	−1.7	0.42	5.17×10^−5^	*RASIP1*	*IZUMO1, FUT1* *FUT2, CA11* *FGF21, FLJ36070*	37
rs225717 (142589792)6q24	C (0.23)	−1.8	0.27	5.99×10^−12^	−1.9	0.60	1.54×10^−3^	−2.2	0.40	7.25×10^−8^	−2.0	0.96	3.70×10^−2^	−1.3	0.50	9.32×10^−3^	*VTA1*	*NMBR*	31
rs10774625 (110394602)12q24	A (0.48)	1.6	0.23	1.16×10^−11^	1.3	0.43	2.50×10^−3^	1.5	0.35	1.82×10^−5^	1.9	0.75	1.10×10^−2^	1.7	0.43	7.70×10^−5^	*ATXN2*	*SH2B3, PTPN11*	9
rs17421627 (87883342)5q14	G (0.08)	2.5	0.43	5.05×10^−9^	2.2	1.16	5.80×10^−2^	1.6	0.63	1.10×10^−2^	5.3	1.91	5.52×10^−3^	3.2	0.71	6.57×10^−6^	-	*MEF2C*	28
rs7824557 (11141521)8p23	G (0.39)	1.4	0.23	2.17×10^−9^	1.6	0.56	4.27×10^−3^	0.8	0.35	2.2×10^−2^	1.7	0.84	4.30×10^−2^	2.2	0.43	5.32×10^−7^	-	*XKR6, PINX1* *SOX7, MTMR9* *GATA4*	69

CHARGE: Cohorts for Heart and Aging Research in Genomic Epidemiology consortium; AGES: Age Gene/Environment Susceptibility – Reykjavik Study; ARIC: Atherosclerosis Risk in Communities Study; CHS: Cardiovascular Health Study; RS: Rotterdam Study; SNP: single nucleotide polymorphism; MAF: minor allele frequency; Beta: Change in retinal venular calibre for each copy of the minor allele; SE: standard error.

No genome-wide significant locus was identified for retinal arteriolar caliber and only one SNP was associated with retinal arteriolar caliber at a significance threshold between 5.0×10^−8^ and 1.0×10^−6^. The QQ-plot (Figure S1B) showed a departure from the line of identity at approximately p<1.0×10^−4^. Figure 1B displays the minus log-transformed p-values for the individual SNPs against their genomic position. The most significant signal was on chromosome 13q12 (rs2281827, per minor allele (T) copy 1.0 µm (SE: 0.21) increase in arteriolar caliber; minor allele frequency (MAF): 0.23; p = 3.53×10^−7^). This signal on chromosome 13q12 was located in *FLT1*, also known as vascular endothelial growth factor receptor.

### Replication in independent cohorts

Table 3 shows the results within each replication cohort for the five loci that were genome-wide significant in the discovery phase. Minor allele frequencies in the replication cohorts were very similar to that in the discovery cohorts. Four out of the five loci showed consistent effects in the combined analyses of the replication cohorts at a Bonferroni-corrected significance threshold of p<0.01 (0.05/5, as five loci were tested in the replication phase), the exception was rs7824557 (8p23). The combined analyses of the discovery and replication cohorts yielded an overall p-value of 1.61×10^−25^ for rs2287921 (19q13). The corresponding values for the other loci were p = 1.25×10^−16^ for rs225717 (6q24), p = 2.15×10^−13^ for rs10774625 (12q24) and p = 7.32×10^−16^ for rs17421627 (5q14). Finally, for rs7824557 (8p23) the overall p-value did not reach genome-wide significance (p = 3.80×10^−7^).

**Table 3:** Results for the five loci associated with retinal venular caliber for the discovery, replication, and combined cohorts.

	*Discovery cohorts combined*			*Replication cohorts* *Individually*												*Replication cohorts combined*			*Discovery and replication cohorts combined*		
	*CHARGE*			*Australian Twins Study*			*UK Twins Study*			*BDES*			*BMES*								
SNP (locus)	Beta	SE	P-value	Beta	SE	P-value	Beta	SE	P-value	Beta	SE	P-value	Beta	SE	P-value	Beta	SE	P-value	Beta	SE	P-value
rs2287921(19q13)	−2.0	0.23	3.30×10^−18^	−1.3	0.72	7.10×10^−2^	−4.2	1.29	1.00×10^−3^	−1.6	0.61	8.30×10^−3^	−4.6	0.93	8.22×10^−7^	−2.3	0.40	6.70×10^−9^	−2.1	0.20	1.61×10^−25^
Rs225717(6q24)	−1.8	0.27	5.99×10^−12^	−2.2	0.84	1.00×10^−2^	−0.3	1.51	8.35×10^−1^	−2.6	0.71	2.00×10^−4^	−1.9	1.07	7.30×10^−2^	−2.1	0.46	3.53×10^−6^	−1.9	0.23	1.25×10^−16^
rs10774625(12q24)	1.6	0.23	1.16×10^−11^	1.6	0.72	3.00×10^−2^	−0.1	1.32	9.59×10^−1^	0.5	0.61	3.75×10^−1^	2.6	0.93	5.55×10^−3^	1.2	0.40	3.33×10^−3^	1.5	0.20	2.15×10^−13^
rs17421627(5q14)	2.5	0.43	5.05×10^−9^	3.5	1.29	7.16×10^−3^	7.4	2.73	7.00×10^−3^	3.3	1.21	6.20×10^−3^	7.1	1.61	1.11×10^−5^	4.5	0.74	1.73×10^−9^	3.0	0.37	7.32×10^−16^
rs7824557(8p23)	1.4	0.23	2.17×10^−9^	−1.4	0.72	4.70×10^−2^	−0.1	1.28	9.22×10^−1^	0.6	0.64	3.85×10^−1^	0.9	0.97	3.44×10^−1^	−0.1	0.41	8.36×10^−1^	1.0	0.20	3.80×10^−7^

CHARGE: Cohorts for Heart and Aging Research in Genomic Epidemiology consortium; BDES: Beaver Dam Eye Study; BMES: Blue Mountains Eye Study; SNP: single nucleotide polymorphism; Beta: Change in retinal venular caliber for each copy of the minor allele; SE: standard error.

**Figure 2:**
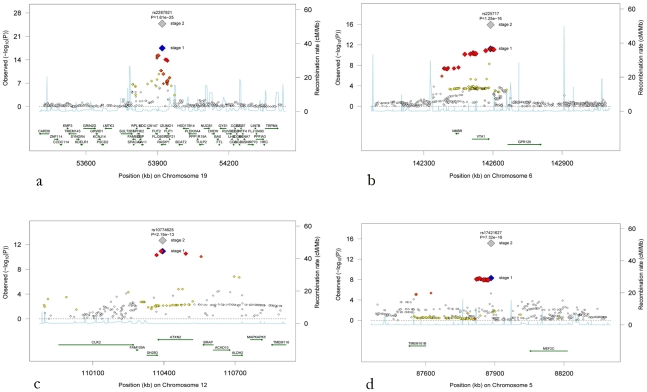
Regional association plots for the four novel loci. (a) Chromosome 19q13, (b) chromosome 6q24, (c) chromosome 12q24, and (d) chromosome 5q14. The blue diamonds show stage 1 p-values (discovery phase) for the top SNP at each locus, whereas the grey diamonds show the p-values following stage 2 meta-analysis including the replication cohorts for that top SNP. P-values from stage 1 for additional SNPs at each locus are colour-coded according to their linkage disequilibrium with the top SNP as follows: r^2^<0.2 white, 0.2<r^2^<0.5 yellow, 0.5<r^2^<orange-red, r^2^>0.8 red.

The regional association plots for these four loci are presented in Figure 2A–2D. After additional adjustments for hypertension and diabetes mellitus, the associations between the four replicated loci and retinal venular caliber remained the same (Table S1).

### Associations with cardiovascular diseases

**Figure 3:**
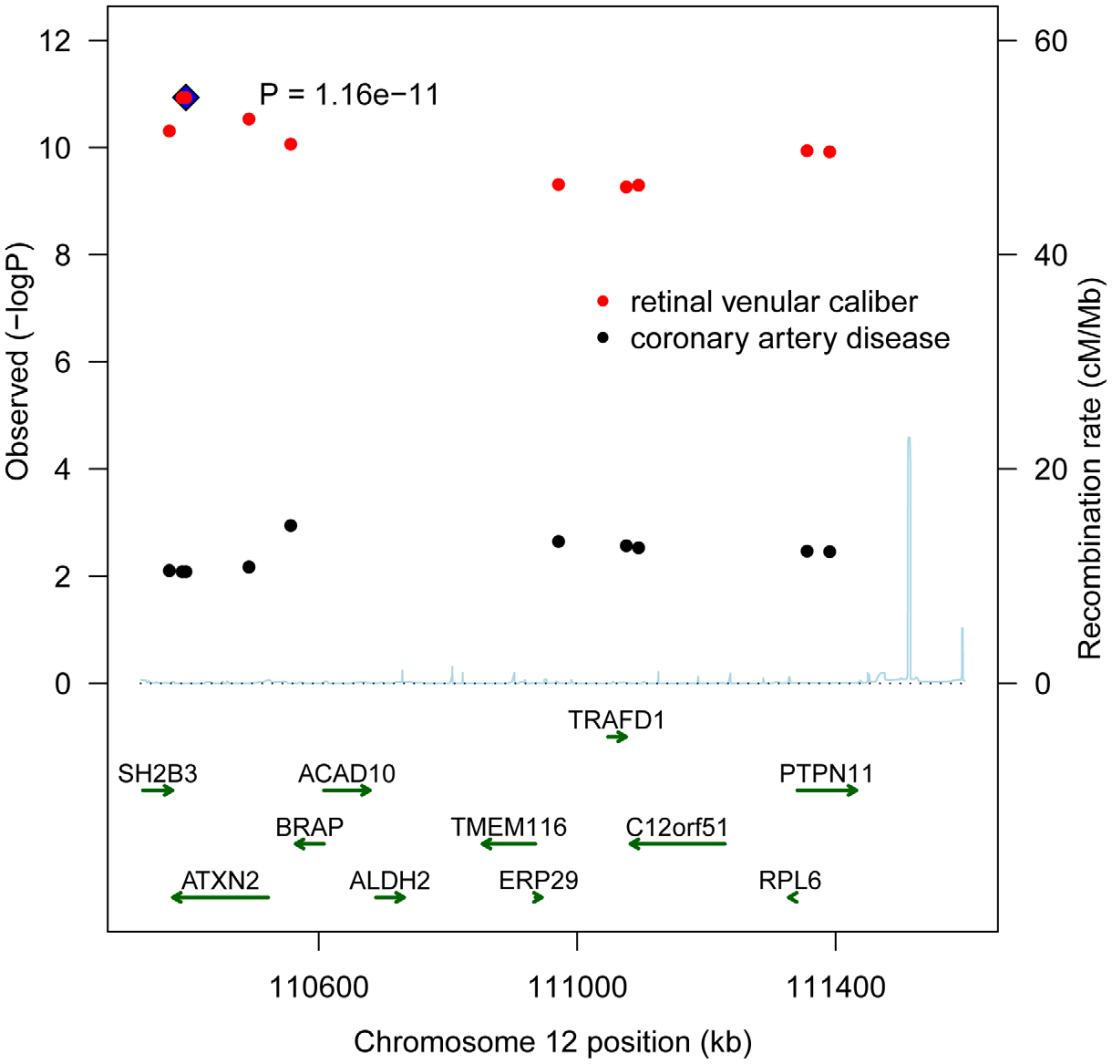
A combined regional association plot showing p-values from CHARGE for the 10 SNPs on 12q24 for retinal venular caliber and from WTCCC for coronary artery disease.

Table 4 presents the results with clinical cardiovascular diseases for the four loci that were successfully replicated in the replication cohorts. These association results provided evidence for 12q24 as a risk locus for coronary artery disease and hypertension. The allelic odds ratios of rs10774625 were 1.13 (95% confidence interval (CI): 1.03–1.24; p = 0.008) for coronary artery disease and 1.06 (95% CI: 1.01–1.12; p = 0.019) for hypertension. As we found the most convincing evidence for rs10774625 to be associated with coronary artery disease, we examined the association with coronary artery disease for all 10 SNPs on locus 12q24 that were genome-wide significant in the current analysis with retinal venular caliber. Figure 3 shows a plot in which the p-values for these 10 SNPs from the current analysis are combined with those for coronary artery disease from WTCCC. We found that all 10 SNPs were significantly associated with coronary artery disease at a nominal p-value of 0.05 suggesting a strong overlap between the association signals of retinal venular caliber and coronary artery disease.

**Table 4:** The association between the four novel loci and cardiovascular diseases.

	*CHARGE* *(CRVE)*	*WTCCC* *(CAD)*	*HVH* *(stroke)*	*HVH* *(MI)*	*Global BPgen* *(HTN)*	DIAGRAM+(DM)
12q24(rs10774625)M.A.: A	Beta: 1.6(SE 0.23)P = 1.16×10^−11^	**OR: 1.13** **(1.03; 1.24)** **P = 0.008**	OR: 1.03(0.89; 1.20)P = 0.66	OR: 1.05(0.94; 1.18)P = 0.39	**OR: 1.06** **(1.01; 1.12)** **P = 0.019**	OR: 1.02(0.98; 1.06)P = 0.36
19q13 (rs2287921)M.A.: T	Beta: −2.0(SE 0.23)P = 3.30×10^−18^	OR: 0.95(0.87; 1.05)P = 0.303	OR: 0.90(0.77; 1.06)P = 0.20	OR: 0.91(0.81; 1.03)P = 0.13	OR: 1.01(0.96; 1.07)P = 0.67	OR: 1.01(0.97; 1.05)P = 0.72
6q24(rs225717)M.A.: C	Beta: −1.8(SE 0.27)P = 5.99×10^−12^	OR: 0.98(0.89; 1.08)P = 0.65	OR: 1.11(0.93; 1.32)P = 0.24	OR: 1.12(0.98; 1.27)P = 0.11	OR: 0.98(0.93; 1.04)P = 0.55	OR: 0.98(0.93; 1.02)P = 0.33
5q14(rs17421627)M.A.: G	Beta: 2.5(SE 0.43)P = 5.05×10^−9^	OR: 1.02(0.88; 1.18)P = 0.81	OR: 1.15(0.83; 1.59)P = 0.39	OR: 1.02(0.79; 1.31)P = 0.89	OR: 1.07(0.98; 1.17)P = 0.14	OR: 0.98(0.91; 1.05)P = 0.60

CHARGE: Cohort for Heart and Aging Research in Genomic Epidemiology Consortium CRVE: central retinal venular equivalent (CRVE), SE: standard error; WTCCC: Wellcome Trust Case Control Consortium; CAD: coronary artery disease; HVH: Heart and Vascular Health Study; MI: myocardial infarction; Global BPgen: Global Blood Pressure Genetics Consortium; HTN: hypertension; DIAGRAM+: Diabetes Genetics Replication and Meta-analysis+; DM: diabetes mellitus; M.A.: Minor allele within CHARGE; OR: odds ratio (with corresponding 95% confidence interval) per copy of the minor allele.

## Discussion

In this meta-analysis of GWAS data from four populations on retinal microcirculation and subsequent replication in four independent cohorts, we identified four novel loci on chromosomes 19q13, 6q24, 12q24 and 5q14 that were consistently associated with retinal venular caliber in persons of Caucasian descent at genome-wide significance of <5.0×10^−8^. The most significant SNPs at each of the four loci were associated with an approximate 2.0µm change in retinal venular caliber for each copy of the minor allele. Locus 12q24 was also associated with coronary heart disease and hypertension. We did not find any loci that reached genome-wide significance for retinal arteriolar caliber, and only one SNP reached highly suggestive levels.

Our study is the first large study to evaluate common genetic variants of the microcirculation, increasingly thought to play a substantial role in the pathogenesis and development of clinical cardiovascular diseases, including coronary heart disease and stroke. The retinal vasculature provides a non-invasive direct view of the human microcirculation. Retinal venular caliber has been shown to predict a range of subclinical [5] and clinical cardiovascular disease [6]. In a recent meta-analysis, wider retinal venules were associated with a hazard ratio of 1.16 (95% CI: 1.06–1.26) for coronary artery disease in women while controlling for other known cardiovascular risk factors [11]. Furthermore, wider venular caliber predicted risk of stroke and is associated with progression of cerebral white matter lesions [10], [12]. Both systemic and environmental factors likely induce variation in retinal venular caliber along with individual genetic differences [5], [6], [17]–[20]. Wider retinal venular caliber has been hypothesized to reflect the effects of hypoxia [32], and an increased nitric oxide production and release of cytokines resulting from activated endothelial cells [33]. This is supported by clinical and epidemiological studies showing larger venular caliber to be associated with systemic biomarkers of inflammation, including C-reactive protein and interleukin-6, and with impaired fasting glucose metabolism, dyslipidemia, obesity and cigarette smoking [5], [34].

The most significant SNP associated with retinal venular caliber was in the *RASIP1* gene (rs2287921, p = 1.61×10^−25^) on chromosome 19q13. *RASIP1* belongs to the family of *RAS* molecules, which have recently been implicated in animal models to be involved in vascular development, endothelial cell migration, capillary tube assembly, blood vessel homeostasis and vascular permeability [35]. Specifically, *RASIP1* is expressed in the endothelium of the developing blood vessels and is essential for proper endothelial cell angiogenic assembly and migration [35].

On chromosome 6q24, the top SNPs were located in or adjacent to *VTA1* and *NMBR* genes. *VTA1* encodes a protein involved in trafficking of the multivesicular body, an endosomal compartment involved in sorting membrane proteins for degradation in lysosomes [36]. Neuromedin B (*NMB*) is a peptide that acts by binding to a specific receptor protein (*NMBR*) and is involved in a number of physiological processes including immune defense, thyroid, adrenocortical function and cognition. *NMB* is also aberrantly expressed by a variety of cancers and is involved in tumor cell proliferation [37].

The signals for association on chromosome 12q24 were spread across a large 1 Mb LD block, including genes such as *SH2B3*, *ATXN2* and *PTPN11*. The most significant SNP was located in *ATXN2*. Defects in the *ATXN2* are the cause of spinocerebellar ataxia type 2 (SCA 2), which belongs to the autosomal cerebellar ataxias characterized by cerebellar ataxia, optic atrophy, ophthalmoplegia and dementia. SCA 2 is caused by extension of a CAG repeat in the coding region of this gene. Another gene in this region is *SH2B3*, which is expressed by vascular endothelial cells and regulates growth factor and cytokine receptor-mediated pathways implicated in lymphoid, myeloid and platelet homeostasis [38]. Our study showed that the most significant SNP in the *SH2B3* region was rs3184504 (p = 4.88×10^−11^). Interestingly, this variant is associated with type 1 diabetes mellitus, a disease in which the risk of developing complications was found to be associated with wider retinal venular caliber [38]. Recent GWAS studies have shown that several SNPs at the locus 12q24 (e.g. rs11065987 in *ATXN2* and rs11066301 in *PTPN11*) are associated with platelet count, hemoglobin concentration, hematocrit, and blood pressure [39]–[41]. Furthermore, replication in independent case-control series including 9,479 cases and 10,527 controls have shown odds ratios of 1.14 (95% CI: 1.10–1.20; p = 2.52×10^−9^) and 1.15 (95% CI: 1.10–1.20; p = 7.05×10^−11^) per minor allele copy for the association of these two SNPs with coronary artery disease [39]. The corresponding allelic odds ratios for myocardial infarction were 1.17 (95% CI: 1.11–1.22; p = 3.43×10^−10^) and 1.18 (95% CI: 1.12–1.23; p = 2.42×10^−12^) [39]. In our discovery cohort, apart from rs10774625 we found nine additional SNPs in the region that were genome-wide significant, including both rs11065987 and rs11066301 (1.5 increase in venular caliber per minor allele for both) that have also been shown to be associated with coronary heart disease and myocardial infarction. Finally, in the present study the association results from WTCCC and Global BPgen confirmed locus 12q24 to be a risk locus for both coronary artery disease and hypertension. Specifically, we found a strong overlap between the association signals of retinal venular caliber and coronay artery disease.

The most significant SNPs at the 5q14 locus were located in an intergenic region. The closest gene in this region is *MEF2C*, which is located about 200 kb downstream. Myocyte enhancer factor 2 (*MEF2*) family proteins are key transcription factors, consisting of four members *MEF2A*, *MEF2B*, *MEF2C* and *MEF2D*, controlling gene expression in myocytes, lymphocytes, and neurons. *MEF2* also plays an important role in cardiogenesis, epithelial cell survival and maintenance of blood vessel integrity. Knockout of *MEF2C* gene in mice is embryologically lethal due to failure in cardiac development [42].

We did not find any loci that reached genome-wide significance for retinal arteriolar caliber. It is possible that genetic factors play a smaller role in arteriolar caliber, which is strongly associated with increasing age and blood pressure [5]–[8]. It is also possible that multiple genetic loci determine retinal arteriolar caliber and each locus exerts only a very weak association that is not detectable using our current study sample size. Thus, in order to examine genetic associations with retinal arteriolar caliber more fully, we are currently in the process of building collaborations with several other studies to increase the sample size of the discovery cohort.

While we have identified four loci associated with retinal venular caliber, the identified SNPs may not represent the causal variants but could be in high linkage disequilibrium (LD) with the causal variants, which remain to be discovered. Further fine mapping of this genomic region will be required to facilitate expression and translational studies. Our study suggests that the effect of common genetic variants on retinal vascular caliber is small, and explain only a small proportion of the heritability of these traits [43]. It remains possible that low frequency variants might be important, but GWAS provides poor coverage of rare variants. With the study populations of predominantly Caucasian descent and stringent checks for latent population substructure, the associations are unlikely to be due to population stratification.

To conclude, our population-based GWAS demonstrate four novel loci on chromosomes 19q13 (within the *RASIP1* locus), 6q24 (adjacent to the *VTA1* and *NMBR* loci), 12q24 (in the region of the *SH2B3*, *ATXN2* and *PTPN11* loci) and 5q14 (adjacent to the *MEF2C* locus) associated with retinal venular caliber, an endophenotype of the microcirculation associated with clinical cardiovascular disease. Furthermore, locus 12q24 was also associated with coronary heart disease and hypertension. While further studies are needed to determine the causal genetic variants that underlie the heritability of this endophenotype, our findings will help focus research on novel genes and pathways involving the microvasculature and its role in the pathogenesis and development of cardiovascular disease.

## Materials and Methods

### Ethics statement

Each cohort secured approval from their respective institutional review boards, and all participants provided written informed consent in accordance with the Declaration of Helsinki.

### Consortium organization

The CHARGE consortium included large prospective community-based cohort studies that have genome-wide marker data and extensive data on multiple phenotypes [21]. All participating studies approved guidelines for collaboration, and a working group arrived at a consensus on phenotype harmonization, covariate selection and analytic plans for within-study analyses and meta-analyses of results.

### Setting

Details of cohort selection, risk factor assessment and retinal vascular caliber measurements in the four studies have been described in Text S1, section 1 [11], [22]–[25]. The AGES is a prospective study with subject recruitment from 2002–2006 of 5,764 surviving members, aged 66 years and older, of the established Reykjavik Study, a cohort of 19,381 participants assembled in 1967 to study cardiovascular disease and its risk factors among those born between 1907 and 1935 [22]. The ARIC study enrolled 15,792 men and women (including 11,478 non-Hispanic whites) from four U.S. communities to investigate the etiology and sequelae of atherosclerosis and cardiovascular risk factors [23]. Participants were between age 45 and 64 years at their baseline examination in 1987–1989. The CHS enrolled 5,888 adults 65 years and older from four field centers to study coronary artery disease and stroke. The baseline examination took place either in 1989–90 or 1992–93 [24]. The Rotterdam Study enrolled 7,983 inhabitants from a district of Rotterdam aged 55 years and older to study cardiovascular, neurological, ophthalmic and endocrine diseases. The baseline examination was in 1990–93 [25].

### Study population

The AGES and Rotterdam cohorts consisted predominantly of Caucasian whites. Only non-Hispanic white participants were included from the ARIC and CHS. Retinal photographs were obtained from participants at the third examination in ARIC and the tenth in CHS. Participants were excluded if their photographs could not be graded (due to cataract, corneal opacities or poor focus) or if genotyping data were unavailable (Table 1).

### Retinal vascular caliber measurements

Retinal vascular caliber was measured using standardized protocols and software that were developed initially at the University of Wisconsin and used in the ARIC study and the CHS, and following slight modifications, also in the Rotterdam and AGES studies (Text S1, section 2) [4], [5], [9], [11], . In brief, participants underwent retinal photography and optic disc-centered images were used to measure vascular caliber. Pharmacological mydriasis was used in the AGES and Rotterdam studies. For ARIC, CHS and Rotterdam the photographs of one eye were digitized using a high-resolution scanner and for the AGES study, photographs of both eyes were captured digitally. All digital retinal images were analyzed with a semi-automated retinal vessel measurement system and the calibers of all retinal arterioles and venules were measured in an area between half and one disc-diameter from the optic disc margin. The Parr-Hubbard-Knudtson formulae were used to compute summary measures for retinal arteriolar and venular calibers in micrometers (µm) and referred to as the “central retinal arteriolar and venular equivalents” [44]. Quality control (QC) measures for intergrader and intragrader intraclass correlation coefficients for retinal vascular calibers for each of the cohorts ranged from 0.76–0.99 in AGES, 0.69–0.89 in ARIC, 0.67–0.91 in CHS to 0.67–0.95 in the Rotterdam Study [4], .

### Genotyping

The consortium was formed after the individual studies had finalized their GWAS platform selection. The four studies included used different platforms: the Affymetrix GeneChip SNP Array 6.0 for the ARIC study, Illumina HumanCNV370-Duo for the AGES study and the CHS and the Illumina Infinium HumanHap550-chip v3.0 for the Rotterdam Study. All studies used their genotype data to impute to the 2.2 million non-monomorphic, autosomal, SNPs identified in HapMap (CEU population). Extensive QC analyses were performed in each cohort (Text S1, sections 3 and 4) [21].

### Statistical analyses within discovery cohorts

Based on an *a priori* analysis plan, each study fitted an additive genetic model with a 1-degree of freedom trend test relating the retinal arteriolar or venular caliber to genotype dosage (0–2 copies of the minor allele) for each SNP, adjusting for age and sex. For the CHS and ARIC studies, the analyses were additionally adjusted for study site. We used linear regression models to calculate regression coefficients (beta) and their standard errors (SE) using the ProbABEL program (http://mga.bionet.nsc.ru/~yurii/ABEL/) in AGES, ARIC and Rotterdam study and the R software in CHS (http://www.r-project.org). Genomic control correction was applied in each study prior to the meta-analysis. To implement genomic control, the λ_gc_ value was used to correct the SE as follows: SE_corrected = SE×√λ_gc_. All four cohorts showed low dispersion with inflation factors in the range of 1.030–1.071.

### Meta-analysis

We conducted a meta-analysis of the beta estimates obtained from the linear regression models from the four cohorts using an inverse-variance weighting using the R software (MetABEL) (Text S1, section 5) [45]. Strand information was available from all cohorts, facilitating the meta-analysis. After QC, filtering, and imputation within each study, we restricted our meta-analysis to the 2,194,468 autosomal SNPs that were common to all cohorts. We decided *a priori* on a genome-wide significance threshold of p<5.0×10^−8^ which corresponds to a p-value of 0.05 with Bonferroni correction for one million independent tests. For 2.2 million tests, it corresponds to an expectation of only 0.11 false positives, regardless of test-dependence [46]. Use of this threshold is also supported by LD patterns observed in deep sequencing work within European populations [47].

### Replication analyses

The genome-wide significant SNPs for each locus from the discovery phase were examined in four replication cohorts. The four replication sample sets included 1,709 participants from the Australian Twins Study, 1,132 from the UK Twins Study, 2,501 from the BDES and 1,310 from the BMES. Retinal vascular caliber measurements used the same methodology and formulas as in the CHARGE cohorts. Details of this and the procedures for genotyping are described in the Text S1, sections 1 and 2. In brief, in the Australian Twins Study, genotyping was performed on the Illumina Human Hap610W Quad array. In the UK Twins Study, 56% of the participants were genotyped using the Illumina 317k HumanHap duo array, whereas the remaining 44% using the Illumina HumanHap610Quad array. In the BDES, SNPs were genotyped using TaqMan SNP genotyping assays (Applied Biosystems, CA). Finally, in the BMES genotyping was performed using the Illumina 610K array.

### Analyses with cardiovascular diseases

In order to examine the association between SNPs that were successfully replicated in the current study and cardiovascular diseases, we obtained association statistics for each of these SNPs from several GWA studies. We obtained these data from the WTCCC on 2000 cases with coronary artery disease and 3000 controls [3], from HVH Study on 501 cases with stroke [28], 1,172 cases with myocardial infarction and 1,314 controls [29], from Global BPgen on 8,871 cases with hypertension and 9,027 controls [30], and from DIAGRAM+ on 8,130 cases with diabetes mellitus and 38,987 controls [31]. Details of each these studies have been described in the Text S1, section 6.

## Supporting Information

Figure S1 Quantile-quantile (QQ)-plot showing the minus log-transformed observed versus the expected p-values after meta-analysis for (A) retinal venular and (B) arteriolar caliber.
(0.26 MB TIF)

Table S1 The association between the top SNPs per genome-wide significant locus and retinal vascular caliber additionally adjusted for diabetes mellitus and hypertension.
(0.05 MB DOC)

Text S1 Sample selection, retinal vascular caliber measurements, genotyping quality control filters and imputation, screening for latent population substructure, meta-analysis techniques, analyses with cardiovascular diseases, reference list.
(0.09 MB DOC)
