# Correction: Four Novel Loci (19q13, 6q24, 12q24, and 5q14) Influence the Microcirculation *In Vivo*

**DOI:** 10.1371/annotation/841bfadf-85d1-4059-894f-2863d73fa963

**Published:** 2010-11-19

**Authors:** M. Kamran Ikram, Xueling Sim, Richard A. Jensen, Mary Frances Cotch, Alex W. Hewitt, M. Arfan Ikram, Jie Jin Wang, Ronald Klein, Barbara E. K. Klein, Monique M. B. Breteler, Ning Cheung, Gerald Liew, Paul Mitchell, Andre G. Uitterlinden, Fernando Rivadeneira, Albert Hofman, Paulus T. V. M. de Jong, Cornelia M. van Duijn, Linda Kao, Ching-Yu Cheng, Albert Vernon Smith, Nicole L. Glazer, Thomas Lumley, Barbara McKnight, Bruce M. Psaty, Fridbert Jonasson, Gudny Eiriksdottir, Thor Aspelund, Global BPgen Consortium, Tamara B. Harris, Lenore J. Launer, Kent D. Taylor, Xiaohui Li, Sudha K. Iyengar, Quansheng Xi, Theru A. Sivakumaran, David A. Mackey, Stuart MacGregor, Nicholas G. Martin, Terri L. Young, Josh C. Bis, Kerri L. Wiggins, Susan R. Heckbert, Christopher J. Hammond, Toby Andrew, Samantha Fahy, John Attia, Elizabeth G. Holliday, Rodney J. Scott, F. M. Amirul Islam, Jerome I. Rotter, Annie K. McAuley, Eric Boerwinkle, E. Shyong Tai, Vilmundur Gudnason, David S. Siscovick, Johannes R. Vingerling, Tien Y. Wong

The second author's name contained an error. Sim Xueling should be Xueling Sim. The correct citation is: Ikram MK, Sim X, Jensen RA, Cotch MF, Hewitt AW, et al. (2010) Four Novel Loci (19q13, 6q24, 12q24, and 5q14) Influence the Microcirculation In Vivo. PLoS Genet 6(10): e1001184. doi:10.1371/journal.pgen.1001184.

