# Supplementary figures and images for: Four Novel Loci (19q13, 6q24, 12q24, and 5q14) Influence the Microcirculation In Vivo
Source: PLoS Genet. 2010 Oct 28;6(10):e1001184. doi: 10.1371/journal.pgen.1001184 (PMC2965750; doi:10.1371/journal.pgen.1001184)

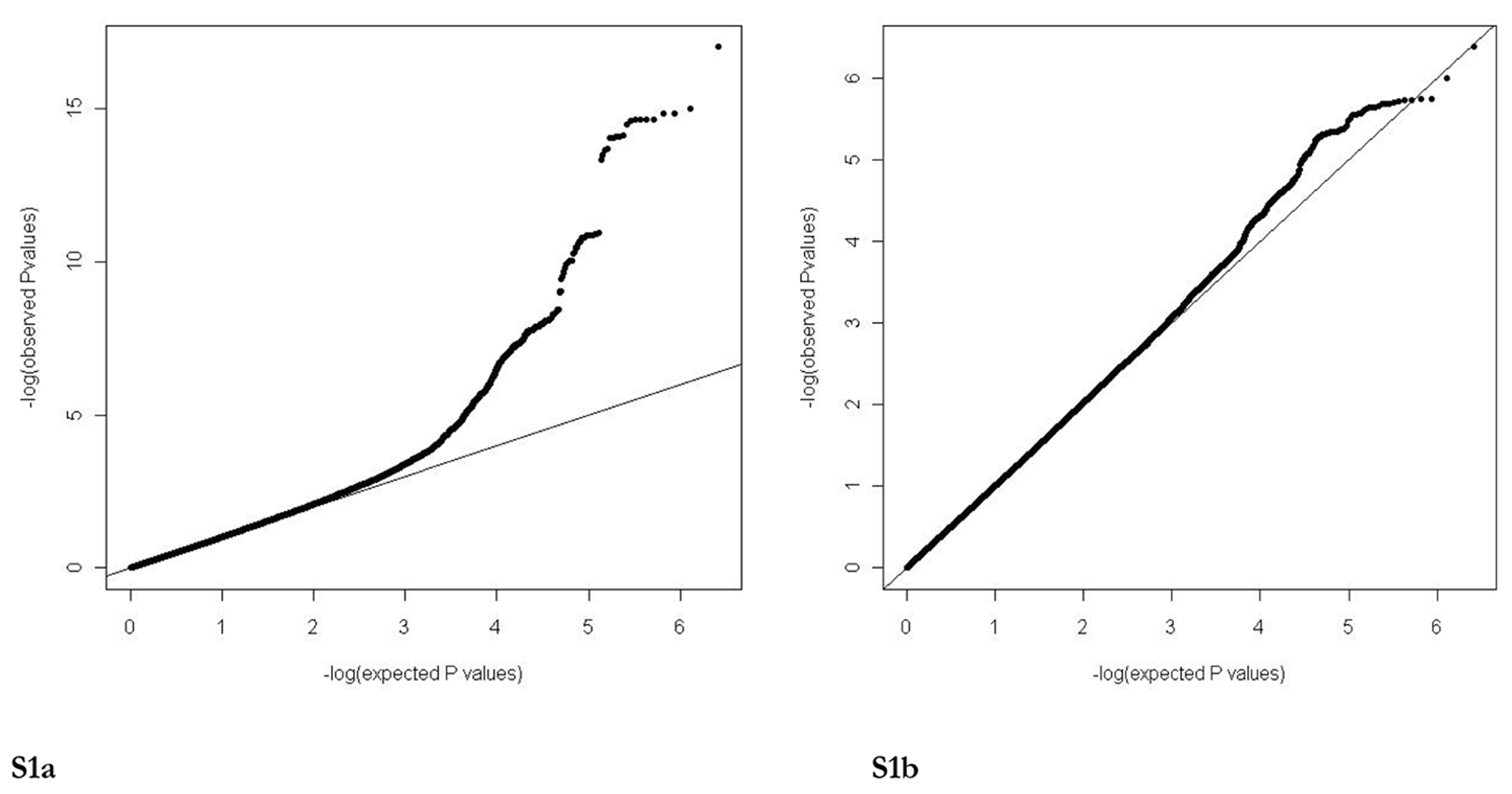

Supplement: Figure S1 — Quantile-quantile (QQ)-plot showing the minus log-transformed observed versus the expected p-values after meta-analysis for (A) retinal venular and (B) arteriolar caliber. (0.26 MB TIF) [file pgen.1001184.s001.tif]
