# Supplementary material for: Four Novel Loci (19q13, 6q24, 12q24, and 5q14) Influence the Microcirculation In Vivo
Source: PLoS Genet. 2010 Oct 28;6(10):e1001184. doi: 10.1371/journal.pgen.1001184 (PMC2965750; doi:10.1371/journal.pgen.1001184)
Supplement: Table S1 — The association between the top SNPs per genome-wide significant locus and retinal vascular caliber additionally adjusted for diabetes mellitus and hypertension. (0.05 MB DOC) [file pgen.1001184.s002.doc]

**Table S1**. The association between the top SNPs per genome-wide significant locus and retinal vascular caliber additionally adjusted for diabetes mellitus and hypertension.

|  | ***Age and sex adjusted*** | ***Age, sex, DM and HT adjusted*** |
| --- | --- | --- |
| **AGES** |  |  |
| 19q13; rs2287921 | -1.0 (SE 0.56); p = 7.40×10^-2^ | -1.0 (SE 0.54); p = 7.61×10^-2^ |
| 6q24; rs225717 | -1.9 (SE 0.60); p = 1.54×10^-3^ | -1.7 (SE 0.58); p = 3.00×10^-3^ |
| 12q24; rs10774625 | 1.3 (SE 0.43); p = 2.50×10^-3^ | 1.3 (SE 0.53); p = 1.69×10^-2^ |
| 5q14; rs17421627 | 2.2 (SE 1.16); p = 5.80×10^-2^ | 2.2 (SE 1.12); p = 4.90×10^-2^ |
| **ARIC** |  |  |
| 19q13; rs2287921 | -2.5 (SE 0.36); p = 3.80×10^-12^ | -2.5 (SE 0.36); p = 1.20×10^-12^ |
| 6q24; rs225717 | -2.2 (SE 0.40); p = 7.25×10^-8^ | -2.2 (SE 0.40); p = 4.24×10^-8^ |
| 12q24; rs10774625 | 1.5 (SE 0.35); p = 2.50×10^-5^ | 1.6 (SE 0.35); p = 7.35×10^-6^ |
| 5q14; rs17421627 | 1.6 (SE 0.63); p = 1.10×10^-2^ | 1.6 (SE 0.62); p = 1.00×10^-2^ |
| **CHS** |  |  |
| 19q13; rs2287921 | -2.9 (SE 0.75); p=1.1x10^-4^ | -2.9 (SE 0.75); p=1.2x10^-4^ |
| 6q24; rs225717 | -2.0 (SE 0.93); p=3.3x10^-2^ | -2.0 (SE 0.93); p=3.4x10^-2^ |
| 12q24; rs10774625 | 1.9 (SE 0.73); p=9.4x10^-3^ | 1.9(SE 0.73); p=9.9x10^-3^ |
| 5q14; rs17421627 | 5.3 (SE 1.9); p=4.2x10^-3^ | 5.2 (SE 1.9); p=5.4x10^-3^ |
| **RS** |  |  |
| 19q13; rs2287921 | -1.7 (SE 0.42); p = 5.17×10^-5^ | -1.7 (SE 0.41); p = 2.08×10^-5^ |
| 6q24; rs225717 | -1.3 (SE 0.50); p = 9.32×10^-3^ | -1.2 (SE 0.49); p = 1.10×10^-2^ |
| 12q24; rs10774625 | 1.7 (SE 0.43); p = 7.70×10^-5^ | 1.7 (SE 0.42); p = 8.75×10^-5^ |
| 5q14; rs17421627 | 3.2 (SE 0.71); p = 6.57×10^-6^ | 3.2 (SE 0.71); p = 7.49×10^-6^ |

AGES: Age Gene/Environment Susceptibility – Reykjavik Study; ARIC: Atherosclerosis Risk in Communities Study; CHS: Cardiovascular Health Study; RS: Rotterdam Study; DM = diabetes mellitus, in AGES, ARIC and CHS defined by self-report, fasting blood glucose ≥ 126 mg/dL (7.0 mmol/L), and/or medication use. In RS, diabetes mellitus was defined as defined as a non-fasting glucose level ≥ 11.0 mmol/l and/or antidiabetic medication use. HT = hypertension; defined by self-report, SBP ≥ 140 and/or DBP ≥ 90 and/or antihypertensive medication use.
