# Supplementary material for: Four Novel Loci (19q13, 6q24, 12q24, and 5q14) Influence the Microcirculation In Vivo
Source: PLoS Genet. 2010 Oct 28;6(10):e1001184. doi: 10.1371/journal.pgen.1001184 (PMC2965750; doi:10.1371/journal.pgen.1001184)
Supplement: Text S1 — Sample selection, retinal vascular caliber measurements, genotyping quality control filters and imputation, screening for latent population substructure, meta-analysis techniques, analyses with cardiovascular diseases, reference list. (0.09 MB DOC) [file pgen.1001184.s003.doc]

**Four Novel Loci (19q13, 6q24, 12q24 and 5q14) Influence the Microcirculation in Vivo**

M.K. Ikram et al.

The Supplementary Appendix has the following sections in order:

1. Sample selection

2. Retinal vascular caliber measurements

3. Genotyping quality control filters and imputation

4. Screening for latent population substructure

5. Meta-analysis techniques

6. Analyses with Cardiovascular Diseases

7. Reference list

**1. Sample selection:**

***Discovery cohorts:***

**Age Gene/Environment Susceptibility – Reykjavik Study (AGES)**

The Reykjavik Study cohort originally comprised a random sample of 30,795 men and women born in 1907-1935 and living in Reykjavik in 1967 [1]. A total of 19,381 persons were enrolled, resulting in a 71% recruitment rate. The study sample was divided into six groups by birth year and birth date within month. One group was designated for longitudinal follow-up and was examined in all stages. One group was designated a control and was not included in examinations until 1991. Other groups were invited to participate in specific stages of the study. Between 2002 and 2006, the AGES study re-examined 5,764 survivors of the original cohort who had participated before in the Reykjavik Study [1]. DNA was genotyped in 3,219 participants using the Illumina 370CNV BeadChip array. Samples were excluded from the dataset based on sample failure, genotype mismatch with reference panel, and sex mismatch. A total of 2,949 participants had data available on genotyping and retinal vessel measurements for these analyses. The study is approved by the Icelandic National Bioethics Committee, VSN: 00-063.

**Atherosclerosis Risk in Communities Study (ARIC)**

The ARIC study is a prospective population-based study of atherosclerosis and clinical atherosclerotic diseases in 15,792 men and women, including 11,478 non-Hispanic white participants, drawn from 4 U.S. communities (Suburban Minneapolis, Minnesota; Washington County, Maryland; Forsyth County, North Carolina, and Jackson, Mississippi) [2]. A detailed study protocol is available on the ARIC study website (http://www.cscc.unc.edu/aric). Clinic examinations included assessment of cardiovascular disease risk factors, a detailed medical and psychosocial history, and measurement of various clinical and laboratory variables. In the first three communities, the sample reflects the demographic composition of the community, whereas the sample from Jackson, Mississippi consisted exclusively of African Americans. Participants were between age 45 and 64 years at their baseline examination in 1987-1989 when blood was drawn for DNA extraction and participants consented to genetic testing [2]. Follow-up examinations took place with an average of three-year interval. Demographic and retinal vessels information were taken from the third examination. Genotyping was performed at the Broad Institute using the Affymetrix Genome-Wide Human SNP Array 6.0. As of June 2008, genotyping had been completed on 8,861 non-Hispanic white participants. Birdseed was used as the genotyping calling algorithm [3]. Samples were excluded on call rate failure (<95%), sex mismatch, cryptic relatedness (1^st^ degree relatives), genotype mismatch with reference panel and outliers based on either IBS clustering or Eigenstrat [4]. Single nucleotide polymorphisms (SNP) with less than 90% call rates or MAF < 1% or HWE p-value <10^-6^ were excluded from imputation.

**Cardiovascular Health Study (CHS)**

The CHS is a population-based cohort study of risk factors for coronary heart disease and stroke in adults aged 65 years or older conducted across four field centers in the United States [5]. The original cohort of 5,201 persons consisting of 4,925 Caucasians was recruited in 1989-1990 from a random sample of people on Medicare eligibility lists and an additional 687 African-Americans were enrolled subsequently for a total sample of 5888. DNA was extracted from blood samples drawn on all participants who consented to genetic testing at their baseline examination in 1989-90. In 2007-2008, genotyping was performed at the General Clinical Research Center's Phenotyping/Genotyping Laboratory at Cedars-Sinai using the Illumina 370CNV Duo BeadChip system. The present report is based upon genotyping data from 1,272 participants, who were free of clinical cardiovascular disease at baseline and had both genotype data and retinal vessel measurements.

**Rotterdam Study**

The Rotterdam Study is a population-based cohort study among inhabitants of a district of Rotterdam (Ommoord), The Netherlands, and aims to examine the determinants of disease and health in the elderly with a focus on neurogeriatric, cardiovascular, bone, and eye disease [6]. All inhabitants aged ≥ 55 years (n = 10,275) were invited and the participation rate was 78%, yielding a total of 7,983 participants. All participants gave written informed consent to retrieve information from treating physicians. Baseline measurements were obtained from 1990 to 1993 and consisted of an interview at home and two visits to the research center for physical examination. Survivors have been re-examined three times: in 1993-1995, 1997-1999, and 2002-2004. All persons attending the baseline examination in 1990-93 consented to genotyping and had DNA extracted. This DNA was genotyped using the Illumina Infinium II HumanHap550chip v3.0 array in 2007-2008 according to the manufacturer’s protocols. Genotyping was attempted in persons with high-quality extracted DNA (n=6,449). From these 6,449, samples with low call rate (<97.5%, n=209), with excess autosomal heterozygosity (> 0.336, n=21), with sex-mismatch (n=36), or if there were outliers identified by the IBS clustering analysis (>3 standard deviations from population mean, n=102 or IBS probabilities > 97%, n=129) were excluded from the study population with some persons meeting more than one exclusion criterion; in total, 5,974 samples were available with good quality genotyping data. Because the baseline ophthalmic part of the Rotterdam Study became operational after the screening of participants had started a total of 4,820 participants had data on genotyping and retinal vessel measurements for the current analyses.

***Replication cohorts:***

**Australian Twins Study**

The Australian Twin Eye Study comprises participants examined as part of the Twins Eye Study in Tasmania (TEST) or the Brisbane Adolescent Twins Study (BATS) [7]. Ethical approval was obtained from the Royal Victorian Eye and Ear Hospital, the University of Tasmania, the Australian Twin Registry and the Queensland Institute of Medical Research. To-date 2,235 people have been reviewed as part of this study, and a total of 950 twin pairs (487 from TEST and 463 from BATS) have been reviewed [7]. Complete phenotype and genetic data are available for 1,709 people. Participants were typed on the Illumina Human Hap610W Quad array, with part of the samples typed alongside the UK Twins Cohort and the remainder typed as a separate contract with DeCODE genetics. The exclusion criteria for SNPs were minor allele frequency ≤1%, Hardy-Weinberg equilibrium (HWE) p<10^-6^, or SNP call rate ≤95% and Illumina Beadstudio Gencall Score < 0.7. Imputation was done with reference to HapMap release 22 CEU using MACH2 (http://www.sph.umich.edu/csg/abecasis/MACH/). Association analysis was performed using Merlin to account for the relatedness of individuals, based on the score test (–fastAssoc option) (http://www.sph.umich.edu/csg/abecasis/merlin/) [8].

**UK Twins Study**

The UK Twins adult twin register is a voluntary cohort comprising 8810 individuals of same-sex pairs, of which, 6,708 individuals have been phenotyped to date [9]. Recruitment originally took place via national media campaigns to study osteoarthritis; the Twins UK cohort is predominantly female with a mean age of 60 years. Retinal images of 1,345 of these individuals were taken between 1998 and 2009, of which the images of 1,236 individuals were suitable for vessel grading analysis. Genotyping information was available for 1,132 of these individuals; 639 of these were genotyped at the Sanger Institute using the Illumina 317k HumanHap duo array and the remaining 493 were genotyped by the Center for Inherited Disease Research (CIDR) using the Illumina HumanHap610Quad array. Of the genotyped individuals, all were female Caucasians aged 59.7 ± 7.7 years (mean ± SD; range, 24–79 years). SNPs from the 610k chip were imputed over the 317k chip to produce a combined dataset from which GW scans were carried out.

**Beaver Dam Eye Study**

The Beaver Dam Eye Study (BDES) is a population based study of age-related eye disease [10]. At the time of census 4926 subjects between 43 and 86 years of age, living in the city of Beaver Dam, Wisconsin, participated in the baseline examination from 1988 to 1990. Informed consent was obtained from the participants following a protocol approved by the Institutional Review Board of the University of Wisconsin. Ninety-nine percent of the participants were Caucasians predominantly from Nothern Europe. A total of 2,579 eligible unrelated participants were considered, of which 2,522 had the measured retinal vessel caliber data available, and therefore served as the participants of this study. The remaining 57 individuals either did not have their retinal vascular caliber measured or they did not have measurable vessels. SNPs were genotyped in 2,522 individuals, on whom vessel measurements were available, using TaqMan SNP genotyping assays (Applied Biosystems, CA) in accordance with manufacturer’s protocol.

**Blue Mountains Eye Study**

The Blue Mountains Eye Study (BMES) is a population-based cohort of a predominantly white population conducted in 1992 [11]. A total of 3,654 residents aged 49 years or older living in two postcode areas, west of Sydney, Australia, participated representing 82.4% of those eligible residents identified in a private census. After 5 years, 2,335 participants (75.1% of survivors) returned for examinations during 1997-99 (BMES II A). A repeat private census was performed in 1999 and identified 1,378 newly eligible residents who moved into the study area or were in the eligible age group. During 1999-2000, 1,174 (85.2%) of them participated in an Extension Study of the BMES (BMES IIB). BMES cross-section II thus includes BMES IIA (66.5%) and BMES IIB (33.5%) participants (n=3,509). DNA was extracted for 3,189 (90.1 %) participants of BMES cross-section II who had blood samples collected. At the time of this report, only 1,421 subjects had genotyping data available, and 1,310 subjects who passed genotyping quality control filters and had retinal vessel measures available were included in the replication analysis.

For the BMES cohort, 1,421 samples were genotyped using the Illumina 610K array. Samples were filtered using PLINK (<http://pngu.mgh.harvard.edu/purcell/plink/)> based on genotype call rate, level of autosomal heterozygosity, evidence of unintended sample duplication, or cryptic relatedness (kinship coefficient >0.1) with other samples. SNPs were filtered based on genotype call rate, minor allele-frequency and deviation from Hardy-Weinberg equilibrium. A total of 1,310 samples passing quality control filters had retinal vessel measures available and were included in association analyses.

Of the five SNPs assessed for replication, one was directly genotyped; genotypes for the remaining four SNPs were imputed using MACH (<http://www.sph.umich.edu/csg/abecasis/MACH/>) version 1.0.16, using phased haplotypes from the HapMap CEU population (Phase II, Release #24) as reference. All imputed SNPs demonstrated high accuracy of imputation (imputation *r^2^* [based on ratio of observed to expected dosage variance] > 0·95). For directly genotyped SNPs, association analyses were conducted using PLINK; for imputed SNPs, association was assessed using the program ProbABEL (<http://mga.bionet.nsc.ru/~yurii/ABEL/>) using the dosage score for the reference allele estimated by MACH. All association analyses were based on a linear (additive) model, adjusting for age and gender.

**2. Retinal vascular caliber measurements:**

**AGES:**

After maximal pharmacologic pupil dilation, retinal images centered on the macula and optic disc of each eye were captured digitally using a 6.3 megapixel Canon CR6 nonmydriatic camera with a Canon D-60 camera back (Canon USA, Inc. Lake Success NY).  Retinal images judged by the trained photographer to be of suboptimal quality were immediately retaken.  Routine feedback on image quality was provided throughout the study after assessment of retinal images by the Ocular Epidemiology Reading Center (Madison WI).  Measurement of retinal vessel caliber was made for all arterioles and venules in the area between 0.5 and 1 disc diameters (DD) from the optic disc margin using a computer-assisted program and a standardized protocol [12]. Measures from individual vessels were combined according to the Parr-Hubbard-Knudtson formulas and summarized as “central retinal arteriolar (CRAE) and venular (CRVE) equivalents” [13]. Inter-grader and intra-grader intra-class correlation coefficients ranged between 0.85-0.95 for CRAE and were 0.99 for CRVE for all graders.

**ARIC:**

The retinal photography was performed at examination 3. Briefly, one 45° nonstereoscopic

color retinal photograph was taken of one eye of each participant using a fundus camera that does not require pharmacologic dilation of the pupil (Canon CR-45UAF; Canon USA, Inc., Lake Success, NY). Photographs were mounted in plastic sheets and sent to a central reading center. To monitor reproducibility of the grading programs, the ARIC Coordinating Center directed the masked replicate grading of selected samples. A Retinal Reading Center technician digitized the color slides at 3175 dots/inch resolution using a 35-mm film scanner (Nikon LS 3510 AF, Tokyo, Japan). Contrast of the retinal vessels against the retinal pigment epithelium was enhanced on a computer workstation (Sun Microsystems, Palo Alto, CA) with a high-resolution 19-inch monitor. The measurement module was custom programmed in Khoros (public domain image processing software from the University of New Mexico - Albuquerque). The retinal measurements were summarized as “CRAE” and “CRVE” (in μm) using the Parr-Hubbard-Knudtson formulas for this study [12,13]. The intragrader and inter-grader intraclass correlation coefficients for CRVE were 0.89 and 0.77 while that of CRAE were 0.69 and 0.74 among three graders.

**CHS:**

Retinal photography procedures in the CHS were similar to the ARIC study. After 5 minutes of dark adaptation, a 45° retinal photograph centered between the optic disc and the macula was obtained of 1 randomly selected eye using a non-mydriatic fundus camera (Canon CR-45UAF; Canon USA, Inc., Lake Success, NY). Photographs were digitized with a high-resolution scanner and evaluated according to a standardized protocol at the Fundus Photograph Reading Center in Madison, WI, by 2 trained and certified graders who were blinded to subject characteristics. For this study, results were summarized as “CRAE” and “CRVE” (in μm) using the Parr-Hubbard-Knudtson formulas [12,13]. In CHS, the intragrader and inter-grader intraclass correlation coefficients for retinal vascular caliber equivalents ranged from 0.67 to 0.91.

**Rotterdam Study:**

Participants underwent a full eye examination at baseline including taking fundus color transparencies of the optic disc (20^0^ field, Topcon Optical Company, Tokyo, Japan) after pharmacological mydriasis. The transparencies from both eyes were digitized with a high-resolution scanner (Nikon LS-4000, Nikon Corporation, Japan), and for each participant the digitized image of one eye with the best quality was analyzed with the Retinal Vessel Measurement System (Retinal Analysis, Optimate, WI; Department of Ophthalmology & Visual Science, University of Wisconsin-Madison). For each participant one summary value was calculated for the arteriolar diameters (CRAE in μm) of the blood column and one for the venular caliber (CRVE) [12,13]. In a random sub-sample of 100 participants we found no statistically significant differences between the right and left eyes for the arteriolar and venular diameters. Four trained graders performed these measurements masked for participant characteristics. Pearson’s correlation coefficients for inter-grader agreement were for CRAE 0.67-0.80 and for CRVE 0.91-0.94. For intra-grader agreement the corresponding figures were 0.69-0.88 and 0.90-0.95.

**Australian Twins Study**

In all subjects following instillation of tropicamide 1%, simultaneous stereoscopic 15^o^ optic disc photographs were taken with a Nidek 3-Dx fundus camera (Nidek, Gamagori, Japan). Images were developed on colour 35 mm slides (Ektachrome, Eastman Kodak, Rochester, NY) and digitized (Nikon CoolScan IV ED slide scanner, Nikon Corp., Tokyo, Japan). Retinal vascular caliber was measured with computer-assisted software (IVAN, University of Wisconsin) according to a standardized protocol and summarized as “CRAE” and “CRVE” [12,13]. Intragrader and intergrader intraclass correlation coefficient was excellent for both CRAE (0.95, 0.93) and CRVE (0.99, 0.98).

**UK Twins Study**

The testing protocol involved dilating pupils with 1% tropicamide and 10% phenylephrine for twins aged over 50 years (n=985); twins who refused dilation (n=25) or those below 50 (n=122) had non-mydriatic images taken. Retinal photographs were taken using a Kowa camera with Kodak Ektachrome 64 film during the period 1998-1999 which were then digitized and analysed by two graders from The Blue Mountains Eye Study using Ivan 1.1 (University of Sydney) [12,13]. This group have previously reported high intergrader reliability for The Blue Mountains Eye Study using quadratic weighted kappa (κ) and correlation analyses (r^2^) for summary of indices of CRAE (κ = 0.85, r^2^ = 0.88), CRVE (κ = 0.90, r^2^ = 0.90) and AVR (κ = 0.75, r^2^ = 0.79). High intragrader correlations for the two graders were also observed of 0.80–0.93 and 0.80–0.92, respectively. Participants phenotyped after 1,999 were photographed using a Nidek AFC210 non-mydriatic digital fundus camera. All images were analysed by one grader using Ivan 1.1 (University of Sydney) where the standard deviation of the differences between repeat measurements was observed as 8.24μm and 5.23μm; the coefficient of variation was observed as 0.35 and 0.63 for CRAE and CRVE, respectively. Measurements were adjusted for pupil dilation, camera and age prior to any genetic analysis.

**BDES**

The eye examination procedure involved taking steroscopic 30^o^ color fundus photographs, centered on the optic disc, of both eyes of participants after dilation. These photographs were then converted to digital images by a high-resolution scanner (Nikon LS2000; Nikon Inc.) A grader identified arterioles and venules and then used a semiautomated procedure to measure the vessel diameters (3.8 μm per pixel) in the area between circles with a 0.5 and 1.0 standard disc diameter, which was a defined unit of measurement (1850μm) established by the Early Treatment Diabetic Retinopathy Study from the optic disc margin. The measurements for the aterioles were combined into CRAE and the measurements for the venules were combined into CRVE, which were robust to variability in vessel number, independent of image scale, and easy to implement using the formulae by Knudtson et al [12,13].

**BMES**

Stereoscopic retinal photographs (30º) were taken at each visit for the macula, optic disc and other retinal fields of both eyes, using a Zeiss FF3 fundus camera (Carl Zeiss, Oberkochen, Germany) after pupil dilation. Gradable retinal photographs of both eyes were obtained from 98% of participants at both baseline and 5-year examinations. The caliber of retinal arterioles and venules from the optic disc photographs at baseline examinations were measured, using a validated computer-assisted method. A digitized grid was placed over the image and all vessels passing completely through a zone between 0.5 to 1 disc diameter from the disc margin were measured. Graders identified each vessel as a venule or arteriole. Using the Retinal Analysis software, also termed Howard program, Optimate, WI; Department of Ophthalmology & Visual Science, University of Wisconsin-Madison five equidistant measures on each vessel and branch were measured in μm. A density pixel histogram showing the width of the central measurement was also displayed, which could be manually adjusted with reference to the image on the screen. The validity of each measurement was judged by evaluating the consistency of the histogram and the visual image, and the correlation between the average and central widths. The Parr-Hubbard formula was used to summarize indices of the average retinal arteriolar and venular diameters in the eye, and referred to as the CRAE and CRVE, respectively [12,13]. Intra- and inter-grader reliability of this method was high, with quadratic weighted kappa values of 0.85 (CRAE) and 0.90 (CRVE) found for inter-grader reliability, and between 0.80-0.93 and 0.80-0.92 for intra-grader reliability of the first and second graders, respectively. Only one eye (mainly the right eye) measurements were used as good correlation was found between measurements of both eyes.

Estimates of CRAE and CRVE obtained from Parr-Hubbard formula were converted into estimates equivalent to measures using a newer computer-assisted program (IVAN program), which was based on 6 largest arterioles and venules, respectively, following a newer formula, termed the Parr-Hubbard-Knudtson formulas.

**3. Genotyping quality control filters and imputation:**

In the discovery cohorts, participant specific quality control filters included filters for call rate, heterozygosity, and number of Mendelian errors per individual. SNP specific quality control filters included filters for call rate, minor allele frequency, Hardy-Weinberg equilibrium, and differential missingness by outcome or genotype (mishap test in PLINK; <http://pngu.mgh.harvard.edu/purcell/plink/)> [14]. The set of genotyped input SNPs used for imputation in each study was selected based on their highest quality GWA data. We used a call rate >95% in CHS and ARIC; >97% in AGES and >98% in Rotterdam; a minor allele frequency > 0.01 in each study; a Hardy-Weinberg p > 1x10^-5^ in CHS and p > 1x10^-6^ in AGES, ARIC, and Rotterdam; and a test of differential missingness by the “mishap” test in PLINK p > 1x10^-9^ in each study. We used either the Markov Chain Haplotyping (MaCH) package (<http://www.sph.umich.edu/csg/abecasis/MACH>)[15] version 1.0.15 software (ARIC and Rotterdam; v1.0.16 for AGES); imputed to plus strand of NCBI build 36, HapMap release #22 (AGES) or BIM-BAM (CHS, imputed to plus strand of NCBI build 35) programs [16]. For each imputed SNP a reliability of imputation was estimated (as the ratio of the empirically observed dosage variance to the expected binomial dosage variance: O/E ratio). For the primary meta-analysis using inverse-variance weighting less weight is given to imputed SNPs with low observed dosage variance (resulting in higher variance of the estimate). For the secondary meta- analysis using the inverse square root (N) weighting the ratio was used to compute an effective sample size.

**4. Screening for latent population substructure**

All four discovery cohorts were screened for latent population substructure (including cryptic relatedness) using suitable programs (i.e, EIGENSTRAT in ARIC and AGES [17,18], an IBD matrix in Rotterdam [19], and using principal component analysis in CHS) and either found no evidence of occult population admixture, or found that the principal components identified were not related to the retinal vascular caliber phenotype.

We studied quantile-quantile (Q-Q) plots to ensure that the p-value distributions in each of the four cohorts conformed to a null distribution at all but the extreme tail. We also calculated the genomic inflation factor (λ_gc_), which measure over-dispersion of test-statistics from association tests indicating population stratification and can be used to apply genomic control [20]. The λ_gc_ for CRVE was 1.071 for AGES, 1.030 for ARIC, 1.063 for CHS, and 1.034 for the Rotterdam Study. For CRAE these values were 1.040 for AGES, 1.031 for ARIC, 1.029 for CHS, and 1.037 for the Rotterdam Study.

**5. Meta-analysis**

After quality control and filtering within each study, the AGES had either genotyped or imputed data for 2,408,991 SNPs, ARIC for 2,557,252 SNPs, the CHS for 2,543,888 SNPs, and the Rotterdam study for 2,543,888 SNPs. We restricted the present meta-analysis to the 2,194,468 autosomal SNPs common to all four studies. Our primary meta-analysis technique was inverse-variance weighting (also known as fixed-effects meta-analysis) after applying genomic control within each individual study. Beta estimates were weighted by their inverse variance and a combined estimate was obtained by summing the weighted betas and dividing by the summed weights. Hence results for SNPs imputed with low certainty were down-weighted because the low reliability-of-imputation (see Section 3) ensures a large variance. In contrast, studies with large sample sizes and with directly genotyped or well-imputed SNPs had a greater effect on the meta-analyses p-value because of small variances. We undertook the meta-analyses at the Rotterdam study using the R-library, MetABEL (http://mga.bionet.nsc.ru/~yurii/ABEL/) [21].

The estimate of λ_gc_ after meta-analysis was 1.007 for CRVE and 1.003 for CRAE indicating no significant inflation of p-values. The quantile-quantile (Q-Q) plot of our inverse variance meta-analysis results for retinal venular diameters (**figure S1A**) and retinal arteriolar diameters (**figure S1B**) show the distribution of the observed test statistic (negative log of p-values, on the *y*-axis) plotted against the distribution of test statistic expected under the null-hypothesis (on the *x*-axis). The observed p-values conform to the null distribution across the entire range until p < 1.0x10^-3^ for CRVE and p < 1.0x10^-4^ for CRAE; we expect that true positive associations reside beyond this region and that population substructure is negligible.

**6. Analyses with Cardiovascular Diseases**

**Wellcome Trust Case Control Consortium (WTCCC)**

The Wellcome Trust Case Control Consortium (WTCCC) was formed with a view to exploring

the utility, design and analyses of GWA studies [22]. It brought together over 50 research groups from the UK that are active in researching the genetics of common human diseases. The WTCCC phase I consisted of seven disease groups, including bipolar disorder (BD), coronary artery disease (CAD), Crohn’s disease (CD), hypertension (HT), rheumatoid arthritis (RA), type 1 diabetes (T1D), and type 2 diabetes (T2D), with 2,000 cases each and 3,000 shared controls. CAD cases had a validated history of either myocardial infarction or coronary revascularization (coronary artery bypass surgery or percutaneous coronary angioplasty) before their 66^th^ birthday. Verification of the history of CAD was required either from hospital records or the primary care physician. Recruitment was carried out on a national basis in the UK through a direct approach to the public via (1) the media and (2) mailing all general practices (family physicians) with information about the study, as previously described [22]. The control samples came from two sources: half from the 1958 Birth

Cohort and the remainder from a new UK Blood Service sample. The latter collection was established specifically for this study and is a UK national repository of anonymized DNA samples from 3,622 consenting blood donors [22]. Imputation was performed using the Markov Chain Haplotyping (MaCH) package (<http://www.sph.umich.edu/csg/abecasis/MACH>) [15] with Hapmap CEU as the reference panel. Logistic regression is then performed, averaging over the dosage score of the reference allele estimated by MACH, using the ProbABEL software (<http://mga.bionet.nsc.ru/~yurii/ABEL/>) with no additional adjustments for covariates.

**The Heart and Vascular Health Study (HVH)**

The Heart and Vascular Health Study (HVH) is a population-based case-control study within Group Health (GH), a large health-maintenance organization in western Washington State. Myocardial Infarction (MI) cases were GH enrollees, 30 to 79 years of age, who had an incident MI between 1995 and 2006, and who were alive at the time of study recruitment. Cases were identified from hospital discharge diagnosis codes and were validated by medical record review as previously described [23]. Stroke cases were GH enrollees, 30 to 79 years of age, who had an incident stroke between 1995 and 2008, and who were alive at the time of study recruitment. Cases were identified from hospital discharge diagnosis codes and were validated by medical record review as previously described [24]. There were 2 sources of participants. The first source included men and women who were on pharmacological therapy for hypertension. The second source included postmenopausal women. Control subjects were a stratified random sample of GH enrollees frequency matched to MI cases on age (within decade), sex, hypertension status, and calendar year of identification. Participants were excluded if they were recent enrollees at GHC, had a history of prior MI or stroke, or if the incident MI or stroke was a complication of a procedure or surgery. All participants provided written informed consent.

**Global Blood Pressure Genetics (Global BPgen) Consortium**

The Global BPgen consortium comprises 17 GWAS studies [25]: the Baltimore Longitudinal Study of Aging (BLSA), British 1958 Birth Cohort (B58C-T1DGC and B58C-WTCCC), Cohorte Lausannoise (CoLaus), Diabetes Genetics Initiative (DGI), European Prospective Investigation of Cancer-Norfolk-Genome Wide Association Study (EPIC-Norfolk-GWAS), Fenland Study, Finland-United States Investigation of NIDDM Genetics (FUSION) study, Invecchiare in Chianti (InCHIANTI), Kooperative Gesundheitsforschung in der Region Augsburg (KORA), the Myocardial Infarction Genetics Consortium (MIGen), Northern Finland Birth Cohort of 1966 (NFBC1966), SardiNIA, Study of Health in Pomerania (SHIP), the Precocious Coronary Artery Disease (PROCARDIS), Supplementation en Vitamines et Mine´raux Antioxydants (SU.VI.MAX) and TwinsUK. We excluded individuals >70 years of age and individuals ascertained on case status for type 1 or 2 diabetes (DGI, FUSION), coronary artery disease (MIgen, PROCARDIS) or hypertension (BRIGHT), leaving 34,433 individuals for analysis. Hypertension was defined by the presence of systolic blood pressure >=140 mm Hg or diastolic blood pressure >=90 mm Hg or self-report of taking a medication for the treatment of hypertension. Normotensive controls were defined as individuals not taking any antihypertensives and having a systolic blood pressure =<120 mm Hg and a diastolic blood pressure =<85 mm Hg. Analysis of hypertension was done using logistic regression to adjust for age, age^2^, sex and body mass index [25].

**Diabetes Genetics Replication and Meta-analysis+ (DIAGRAM+)**

The DIAGRAM+ consortium combined results from eight genome-wide diabetes mellitus type 2 (T2D)-case control association studies involving European-descent samples [26]: DGDG, deCODE, DGI, Rotterdam, EUROSPAN, FUSION, KORAgen and WTCCC. The sample included 8,130 cases and 38,987 controls with an effective sample size of 22,044 (that is, a sample size with power equivalent to 11,022 cases and 11,022 controls). Association analysis was carried out using an additive model on a ln (OR) scale. Genomic control correction of autosomal data was performed from each individual study (separately for directly genotyped and for imputed data) by inflating the standard error of the estimated ln (OR) so the significance of the SNP matched that of the genomic control P-value. For those autosomal SNPs with data on ≥17,000 effective total samples, we estimated the genomic control inflation factor to be 1.074 (1.069 after removing 9,939 SNPs from regions of known T2D association). Inverse variance-weighted meta-analysis to com­bine association results from all studies [26].

**7. Reference List**

1. Harris TB, Launer LJ, Eiriksdottir G, Kjartansson O, Jonsson PV, et al (2007) Age, Gene/Environment Susceptibility-Reykjavik Study: multidisciplinary applied phenomics. Am J Epidemiol 165:1076-1087.
2. The Atherosclerosis Risk in Communities (ARIC) Study: design and objectives (1989) The ARIC investigators. Am J Epidemiol 129:687-702.
3. Korn JM, Kuruvilla FG, McCarrollo SA, Wysoker A, Nemesh J, et al (2008) Integrated genotype calling and association analysis of SNPs, common copy number polymorphisms and rare CNVs. Nat Genet 40:1253-1260.
4. Price AL, Patterson NJ, Plenge RM, Weinblatt ME, Shadick NA, et al (2006) Principal components analysis corrects for stratification in genome-wide association studies. Nat Genet 38:904-909.
5. Fried LP, Borhani NO, Enright P, Furberg CD, Gardin JM, et al (1991) The Cardiovascular Health study: design and rationale. Ann Epidemiol 1:263-276.
6. Hofman A, Breteler MM, van Duijn CM, Janssen HL, Krestin GP, et al (2009) The Rotterdam Study: 2010 objectives and design update. Eur J Epidemiol 24:553-572.
7. Mackey DA, Mackinnon JR, Brown SA, Kearns LS, Ruddle JB, et al (2009) Twins Eye Study in Tasmania (TEST): Rationale and Methodology to Recruit and Examine Twins. Twin Res Hum Genet 12:441-454.
8. Sun C, Zhu G, Wong TY, Hewitt AW, Ruddle JB, et al (2009) Quantitative genetic analysis of the retinal vascular caliber: the Australian Twins Eye Study. Hypertension 54:788-795.
9. Hammond CJ, Snieder H, Spector TD, Gilbert CE (2000) Genetic and environmental factors in age-related nuclear cataracts in monozygotic and dizygotic twins. N Engl J Med 342:1786-1790.
10. Klein R, Klein BE, Linton KL, De Mets DL (1991) The Beaver Dam Eye Study: visual acuity. Ophthalmology 98:1310-1315.
11. Leung H, Wang JJ, Rochtchina E, Tan AG, Wong TY, et al (2003) Relationships between age, blood pressure, and retinal vessel diameters in an older population. Invest Ophthalmol Vis Sci 44:2900-2904.
12. Hubbard LD, Brothers RJ, King WN, Clegg LX, Klein R, et al (1999) Methods for evaluation of retinal microvascular abnormalities associated with hypertension/sclerosis in the Atherosclerosis Risk in Communities Study. Ophthalmology 106:2269-2280.
13. Knudtson MD, Lee KE, Hubbard LD, Wong TY, Klein R, et al (2003)  Revised formulas for summarizing retinal vessel diameters. Curr Eye Res 27:143-149.
14. Purcell S, Neale B, Todd-Brown K, Thomas L, Ferreira MA, et al (2007) PLINK: a tool set for whole-genome association and population-based linkage analyses. Am J Hum Genet 81:559-575.
15. Li Y and Abecasis GR (2006) Mach 1.0: Rapid Haplotype Reconstruction and Missing Genotype Inference. Am J Hum Genet S79:2290.
16. Servin B, Stephens M (2007) Imputation-based analysis of association studies: candidate regions and quantitative traits. PLoS Genet 3:e114.
17. Patterson N, Price AL, Reich D (2006) Population structure and eigenanalysis. PLoS Genet 2:e190.
18. Price AL, Patterson NJ, Plenge RM, Weinblatt ME, Shadick NA, et al (2006) Principal components analysis corrects for stratification in genome-wide association studies. Nat Genet 38:904-909.
19. Richards JB, Rivadeneira F, Inouye M, Pastinen TM, Soranzo N, et al (2008) Bone mineral density, osteoporosis, and osteoporotic fractures: a genome-wide association study. Lancet 371:1505-1512.
20. Bacanu SA, Devlin B, Roeder K (2000) The power of genomic control. Am J Hum Genet 66:1933-1944.
21. Aulchenko YS, Ripke S, Isaacs A, Van Duijn CM (2007) GenABEL: an R library for genome-wide association analysis. Bioinformatics 23:1294-1296.
22. Wellcome Trust Case Control Consortium (2007) Genome-wide association study of 14,000 cases of seven common diseases and 3,000 shared controls. Nature 447:661-678.
23. Psaty BM, Heckbert SR, Koepsell TD, Siscovick DS, Raghunathan TE, et al (1995) The risk of myocardial infarction associated with antihypertensive drug therapies, JAMA 274:620-625.
24. Klungel OH, Heckbert SR, Longstreth WT Jr, Furberg CD, Kaplan RC, et al (2001) Antihypertensive drug therapies and the risk of ischemic stroke, Arch Intern Med 161:37-43.
25. Newton-Cheh C, Johnson T, Gateva V, Tobin MD, Bochud M, et al (2009) Genome-wide association study identifies eight loci associated with blood pressure. Nat Genet 41:666-676.
26. Voight BF, Scott LJ, Steinthorsdottir V, Morris AP, Dina C, et al (2010) Twelve type 2 diabetes susceptibility loci identified through large-scale association analysis. Nat Genet 42:579-589.
